# Increased hippocampal efficiency is associated with greater headache frequency in adolescents with chronic headache

**DOI:** 10.1093/texcom/tgad013

**Published:** 2023-07-15

**Authors:** Karen L Cobos, Xiangyu Long, Catherine Lebel, Nivez Rasic, Melanie Noel, Jillian V Miller

**Affiliations:** Anesthesiology, Perioperative & Pain Medicine, University of Calgary, Calgary, Alberta, Canada; Vi Riddell Children’s Pain & Rehabilitation Centre, Alberta Children’s Hospital, Calgary, Alberta, Canada; Behaviour & the Developing Brain, Alberta Children’s Hospital Research Institute, Calgary, Alberta, Canada; Owerko Centre, Alberta Children’s Hospital Research Institute, Calgary, Alberta, Canada; Brain and Mental Health, Hotchkiss Brain Institute, Calgary, Alberta, Canada; Mathison Centre for Mental Health Research & Education, Hotchkiss Brain Institute, Calgary, Alberta, Canada; Behaviour & the Developing Brain, Alberta Children’s Hospital Research Institute, Calgary, Alberta, Canada; Owerko Centre, Alberta Children’s Hospital Research Institute, Calgary, Alberta, Canada; Brain and Mental Health, Hotchkiss Brain Institute, Calgary, Alberta, Canada; Mathison Centre for Mental Health Research & Education, Hotchkiss Brain Institute, Calgary, Alberta, Canada; Radiology, University of Calgary, Calgary, Alberta, Canada; Behaviour & the Developing Brain, Alberta Children’s Hospital Research Institute, Calgary, Alberta, Canada; Owerko Centre, Alberta Children’s Hospital Research Institute, Calgary, Alberta, Canada; Brain and Mental Health, Hotchkiss Brain Institute, Calgary, Alberta, Canada; Mathison Centre for Mental Health Research & Education, Hotchkiss Brain Institute, Calgary, Alberta, Canada; Radiology, University of Calgary, Calgary, Alberta, Canada; Anesthesiology, Perioperative & Pain Medicine, University of Calgary, Calgary, Alberta, Canada; Vi Riddell Children’s Pain & Rehabilitation Centre, Alberta Children’s Hospital, Calgary, Alberta, Canada; Behaviour & the Developing Brain, Alberta Children’s Hospital Research Institute, Calgary, Alberta, Canada; Anesthesiology, Perioperative & Pain Medicine, University of Calgary, Calgary, Alberta, Canada; Vi Riddell Children’s Pain & Rehabilitation Centre, Alberta Children’s Hospital, Calgary, Alberta, Canada; Behaviour & the Developing Brain, Alberta Children’s Hospital Research Institute, Calgary, Alberta, Canada; Owerko Centre, Alberta Children’s Hospital Research Institute, Calgary, Alberta, Canada; Brain and Mental Health, Hotchkiss Brain Institute, Calgary, Alberta, Canada; Mathison Centre for Mental Health Research & Education, Hotchkiss Brain Institute, Calgary, Alberta, Canada; Psychology, University of Calgary, Calgary, Alberta, Canada; Anesthesiology, Perioperative & Pain Medicine, University of Calgary, Calgary, Alberta, Canada; Vi Riddell Children’s Pain & Rehabilitation Centre, Alberta Children’s Hospital, Calgary, Alberta, Canada; Behaviour & the Developing Brain, Alberta Children’s Hospital Research Institute, Calgary, Alberta, Canada; Owerko Centre, Alberta Children’s Hospital Research Institute, Calgary, Alberta, Canada; Brain and Mental Health, Hotchkiss Brain Institute, Calgary, Alberta, Canada; Mathison Centre for Mental Health Research & Education, Hotchkiss Brain Institute, Calgary, Alberta, Canada; Psychology, University of Calgary, Calgary, Alberta, Canada

**Keywords:** depression, fMRI, hippocampus, imaging, pediatric chronic pain

## Abstract

Adults with chronic headache have altered brain hippocampal efficiency networks. Less is known about the mechanisms underlying chronic headache in youth. In total, 29 youth with chronic headache (10–18 years), and 29 healthy, age- and sex-matched controls tracked their headache attacks daily for 1-month period. Following this, they underwent a resting state functional magnetic resonance imaging scan and self-reported on their pubertal status, post-traumatic stress, anxiety, and depression symptoms. Graph-based topological analyses of brain networks, rendering hippocampal efficiency values were performed. *T*-tests were used to compare hippocampal efficiency metrics between patients and controls. Linear regression was used to examine significant hippocampal efficiency metrics in relation to headache frequency in patients, controlling for age, sex, pubertal status, post-traumatic stress, anxiety, and depression symptoms.

Patients had higher right hippocampal global efficiency, shorter right hippocampal path length, and higher right hippocampal clustering coefficient compared to controls (*P* < 0.05). Higher right hippocampal global efficiency, shorter right hippocampal path length, and higher right hippocampal clustering coefficients were positively associated with greater headache frequency (*P* < 0.05). The hippocampus is largely involved in memory formation and retrieval, and this data provides additional support for previous findings demonstrating the importance of the hippocampus and pain memories for the chronification of pain.

## Introduction

Pediatric chronic headache (headache for 3 months or longer) is highly prevalent, affecting approximately one in four youth (King et al. 2011). If left unmanaged, chronic headache can lead to significant functional disability (Wang et al. 2007), and lower health-related quality of life (Bandell-Hoekstra et al. 2002). Children with chronic headache demonstrate poorer academic performance compared to children without chronic headache (Arruda and Bigal 2012), and are at an increased risk of chronic headache and psychiatric morbidity in adulthood (Fearon and Hotopf 2001). Approximately 65.5% of pediatric patients with chronic headache have at least one psychiatric disorder, with only 36.2% of these patients receiving mental health services (O'Brien and Slater 2016).

Depression, anxiety and post-traumatic stress disorder (PTSD) have been linked to the development and maintenance of chronic pain. Each of these conditions can lead to the activation of the hypothalamic–pituitary–adrenal (HPA) axis and production of stress hormones (cortisol in humans) (Chrousos 2009). Activation of the hypothalamus, leads to the co-release of hormones that stimulate the synthesis and release of adrenocorticotropin (ACTH) from the anterior pituitary gland (Francis et al. 1999; Heim and Nemeroff 2001; Gunnar and Quevedo 2007). ACTH secretion influences the release of glucocorticoids from the adrenal cortex into general circulation. Then, cortisol binds with glucocorticoid receptors in the hippocampus (and other brain regions) to inhibit further production of cortisol (Gunnar and Quevedo 2007). However, prolonged exposure to stress and/or pain can disrupt this cortisol feedback loop. Individuals with chronic pain, PTSD, anxiety and/or depression often demonstrate HPA dysfunction, including altered glucocorticoid negative feedback, and abnormal cortisol levels (Lentjes et al. 1997; Griep et al. 1998; Heim et al. 1998; Boyer 2000; Yehuda 2000; Blackburn-Munro and Blackburn-Munro 2001; Korszun et al. 2002; Tennant and Hermann 2002; Timmers et al. 2019; Nelson et al. 2019). Importantly, the HPA axis regulates the transcription of genes (Chrousos 2009), which therein can impact brain structure and function (Hakamata et al. 2022). The hippocampus is one of the most thoroughly investigated brain regions owing to its known vulnerability to prolonged stress (McEwen et al. 2016).

Over the last few decades, studies have been investigating the biopsychosocial factors underlying headache symptomology and chronicity. Functional magnetic resonance imaging (fMRI) studies have linked the hippocampus and brainstem to the first phase of a headache attack, and the limbic system to the maintenance of headache pain (Hadjikhani et al. 2013; Schulte and May 2016). Graph theory approaches using fMRI data have revealed that chronic headache leads to alterations in functional brain connectivity. Chronic headache has been associated with the disruption of hippocampal networks (Liu et al. 2015). Increased and decreased nodal centralities (number of neighboring co-activated brain regions (Farahani et al. 2019)), have been identified (Zhang et al. 2017). Moreover, a loss of topological organization in cortical networks has been observed in patients with migraine compared to healthy controls (Liu et al. 2012). To our knowledge, only a few studies have investigated whether these alterations in brain networks are conserved among youth with chronic headache (Hashemi et al. 2012; Nahman-Averbuch et al. 2022; Szabo et al. 2022).

Given that chronic headache is a growing epidemic, it is imperative to identify biopsychosocial factors associated with this pain condition in youth. This study examined hippocampal brain efficiency in youth with chronic headache as compared to healthy controls, accounting for comorbid mental health symptomology. We hypothesized that youth with chronic headache would have lower hippocampal nodal efficiency in functional networks as compared to healthy controls. Further, we hypothesized that lower hippocampal nodal efficiency would be associated with increased headache frequency in youth with chronic headache. Understanding the mechanisms that lead to the development and maintenance of chronic headache may help facilitate earlier diagnosis and could lead to the development of evidence based, targeted interventions.

## Methodology

Research ethics approval was obtained from the University of Calgary Conjoint Health Research Ethics Board (REB15-3100), and the study followed the Declaration of Helsinki. Written informed consent was obtained from the parents of all participants. Youth over the age of 14 provided informed written consent, while youth under the age of 14 provided informed written assent.

### Population

This study used a subset of data from a larger study, which included multiple pain conditions to examine the co-occurrence of pediatric pain and mental health issues among youth with chronic pain (Miller et al. 2021). The present study included 30 youth aged 10–18 years with chronic headache, recruited from outpatient multidisciplinary chronic pain programs at the Alberta Children's Hospital from November 2017 to December 2018. New patients, as well as patients that had previously been treated in the chronic pain clinics within the past 2 years were included. Eligibility included headache pain for 3 months or more, with no underlying disease cause. Patients were excluded if they had a neurodevelopmental disorder or psychosis. Age- and sex-matched controls were recruited through community advertisements and the Healthy Infants and Children Clinical Research Program at the University of Calgary, a large database of healthy youth who have consented to be contacted for research. Control subjects were excluded if they had a neurodevelopmental disorder or psychosis, or if they had chronic pain (pain lasting 3 months or more). Data for this study was prospectively collected.

### Headache tracking

For 1 month, on a calendar that was provided, both patients and controls reported daily on the occurrence of their headache or migraine attacks, and medication use. Controls were asked to track their headache attacks as well, because it was expected that they would also experience headache attacks, albeit fewer, given the Alberta climate—Pre-chinook days and high-wind chinook days are associated with an increased risk of migraine in individuals who do not have chronic headache (Cooke et al. 2000). Those who experienced headache or migraine attacks were asked to respond to a supplementary questionnaire regarding the timing, location, symptoms, and functional limitations associated with their attack (i.e. activity level, school performance, chores, etc). This headache calendar and the supplementary questionnaire were originally developed and used for the Childhood and Adolescent Migraine Prevention study (trial registration: NCT01581281), which is a placebo controlled, multicenter, comparative effectiveness study of amitriptyline, and topiramate for the prevention of episodic and chronic migraine in children and adolescents (Hershey et al. 2013).

### Pubertal status, anxiety, depression, and PTSD questionnaires

One-week prior to their MRI, participants completed the following reliable and validated questionnaires.

### Pubertal status

Youth reported on their pubertal status using A Self-Administered Rating Scale for Pubertal Development (Carskadon and Acebo 1993). This is a 5-item scale scored on a 4-point Likert scale ranging from “not yet started” (1) to “seems complete” (4). Point values are averaged across items to derive pubertal status. A validation study comparing mean self-rating scores from the children to a pediatrician’s assessment of Tanner stage based upon pubic hair growth found the Spearman correlation coefficient between self-rated and pediatrician-rated physical development to be high (Carskadon and Acebo 1993). Pubertal status, in addition to age, was used as a covariate in the analyses because research has shown that pubertal maturation has unique and additive influences on structural neurodevelopmental trajectories over and above age (Blakemore et al. 2010; Goddings et al. 2014; Herting and Sowell 2017).

#### Anxiety and depressive symptoms

Participants completed the Revised Child Anxiety and Depression Scale (RCADS) (Chorpita et al. 2015). A total of 47 items are divided into seven subscales including: separation anxiety disorder, phobia, generalized anxiety disorder, panic disorder, obsessive compulsive disorder, and low mood. Each item is rated on a four-point Likert scale. The RCADS questionnaire yields a Total Anxiety Score which is a sum of five anxiety subscales. Total anxiety and low mood scores were included in the analysis. Higher scores are indicative of greater anxiety/depressive symptoms. The RCADS has demonstrated excellent scale validity and reliability—within scale reliability and internal subscale reliability (Esbjørn et al. 2012; Kösters et al. 2015).

#### Post-traumatic stress symptoms

Participants filled out the Child PTSD Symptom Scale-V (CPSS-V) (Foa et al. 2001). It is a 20-item measure that maps onto the Diagnostic and Statistical Manual of Mental Disorders Fifth Edition PTSD criteria (American Psychiatric Association 2013) and assesses post-traumatic stress symptoms (PTSS) experienced by youth in the past month. Both participants and controls were asked to name their most distressing or traumatic memory. Keeping that event in mind, they responded to 20-items assessing PTSS on a five-point Likert scale, which ranged from “not at all” to “six or more times a week/almost always”. The 20 items were summed to obtain the total symptom severity score. Total symptom severity scores range from 0 to 80, with higher scores indicating higher PTSS. A score of 31 or above indicates clinically elevated PTSS . The CPSS-V has demonstrated excellent internal consistency, good test—retest reliability, and good convergent validity (Foa et al. 2001).

### Medical imaging

#### Neuroimaging

Youth underwent MRI scanning using a 32-channel head coil on a GE 3T Discovery MR750w (GE, Milwaukee, WI) system at the Alberta Children’s Hospital. Youth were not sedated and were able to watch a movie of their choosing except during resting state fMRI, during which they were presented a fixation cross and were asked to look at the cross and think of nothing in particular. T1-weighted anatomical images were acquired with FSPGR BRAVO with a flip angle of 10°, 230 slices, repetition time = 6.8 ms, echo time = 3.0 ms, voxel size of 0.8 mm × 0.8 mm × 0.8 mm, and inversion time of 540 ms, and scan duration of 4:50 min. Resting state fMRI data were acquired using a gradient—echo planar imaging sequence with repetition time = 2 s, echo time = 30 ms, flip angle = 60°, 38 slices, voxel size = 3.6 × 3.6 × 3.6 mm, and scan duration of 6:10 min. Including other sequences and potential repeated sequences, the entire protocol lasted 45–60 min. One of the patient’s and one of the control’s scans were terminated early due to excessive movement.

### Image processing

#### Preprocessing

 Resting state fMRI data were pre-processed using Analysis of Functional NeuroImages (AFNI) and the Oxford Centre for fMRI of the Brain Software Library (FSL) (Cox 1996; Jenkinson et al. 2012). The T1-weighted image of each participant was skull stripped and segmented into gray matter, white matter, and cerebrospinal fluid (CSF) images. These images were then co-registered to their own fMRI space. Slice timing and head motion were both corrected. The methods for skull stripping, slice timing, and head motion corrections have been widely applied in previous studies (Welch-McCaffrey 1985; Long et al. 2017). For further study, the averaged relative frame-wise displacement (FD) was determined (Jenkinson et al. 2002). The averaged signals from each whole-brain mask, CSF mask, white matter mask, and the six head motion parameters, as well as their temporal derivatives and quadratic term signals, were used to generate a 36-parameter matrix (Satterthwaite et al. 2013). Volumes with a high relative FD (>0.3 mm) were used to generate a spike matrix (Power et al. 2014). The fMRI data were then regressed using a combination of the 36-parameter matrix and the spike matrix. All datasets had spike volumes ˂4-min in length (i.e. greater than 4-min of low-motion data), and therefore were included for analysis. The remaining fMRI signals were then band-pass filtered (0.009–0.08 Hz), underwent linear-trend removal, were spatially converted to Montreal Neurological Institute standard space using a pediatric template aged 13–18 years (Fonov et al. 2011) and spatially smoothed with a 4 mm full width at half maximum kernel. FSL (Jenkinson et al. 2012) was used to perform slice timing, head motion correction, T1-weighted image segmentation, head motion outlier detection, co-registration, as well as spatial normalization and smoothing. AFNI version AFNI 21.1.20 (Cox 1996) was used to perform regression of the nuisance signals, band-pass filtering, and linear-trend elimination.

#### Measurement of Headmotion

FD is a method for determining whether head movement is detected from one volume to the next. It is calculated by adding the absolute values for the compartmentalized realignment estimates (backwards differences) at every time point, thereby providing an index for head motion (Power et al. 2012; Power et al. 2014). By convention, FD of the first volume of a run is zero.

#### Functional connectome construction

An Automatic Anatomical Labeling atlas (AAL) template was used to subdivide the brain into 90 regions, excluding the cerebellum (Rorden and Brett 2000; Tzourio-Mazoyer et al. 2002; Bullmore and Sporns 2009; Kaiser 2017). Within each AAL regions, the average time series was calculated. Pearson’s correlation coefficients were then computed between the average time series from each AAL regions, and Fischer’s z scores were generated to create a 90 × 90 connectivity matrix for each data set. The graph theoretical measures were computed for the threshold and binarized individual connectivity matrix of the whole time series, similar to previous studies (Wozniak et al. 2017) (Long et al. 2019). The threshold of the matrix was set at *r* = 0.15, *P* < 0.05. This threshold has been used in several previously published studies (Finn et al. 2015; Metzak et al. 2022). By setting this threshold, we removed the correlation coefficients that were not significant from the connectivity matrix. The correlation coefficient is considered significant when equals to 0.15 at *P* < 0.05 with 180 volumes in the correlation analysis. In this step, pseudo-connectivities are removed from the graphic measures. The overall functional connectivity was then measured by calculating the mean of the positive values of the raw individual connectivity matrix; and a two-sample *t*-test was computed to analyze the overall differences in functional connectivity between patients and controls. Utilizing the GRETNA toolbox (Wang et al. 2015), nodal efficiencies were calculated for each binary matrix. Each node’s clustering coefficient is a measure of the number of connections among its neighbors. The shortest path length represents the fastest pathway possible for information to be transferred between two nodes. Local efficiency refers to how efficiently information is being transferred between each node and its neighbors. Global efficiency is a measure of how efficiently information is being transferred between nodes across a graph, with higher efficiency indicating that fewer pathways must be used to reach other nodes. Degree centrality refers to the number of connections with each node (Bassett and Bullmore 2006; Bullmore and Sporns 2009; Rubinov and Sporns 2010; Wang et al. 2010).

### Data analyses

Statistical analyses were performed using IBM SPSS Statistics software version 26 (IBM Corporation). Normality was examined and a few violations were detected. Thus, the following variables were log transformed: (i) anxiety, (ii) depression, and (iii) PTSS. Independent sample *t*-tests and chi-square tests were used to compare sample characteristics. Independent sample *t*-tests were performed to compare hippocampal graph theoretical metrics between patients and controls. Linear regression was then used to examine significant hippocampal efficiency metrics in relation to headache frequency in patients, controlling for child age, sex, pubertal status, PTSS, anxiety, and depression symptoms. The nodal level results were displayed by BrainNet Viewer toolbox (Xia et al. 2013).

## Results

**Fig. 1:**
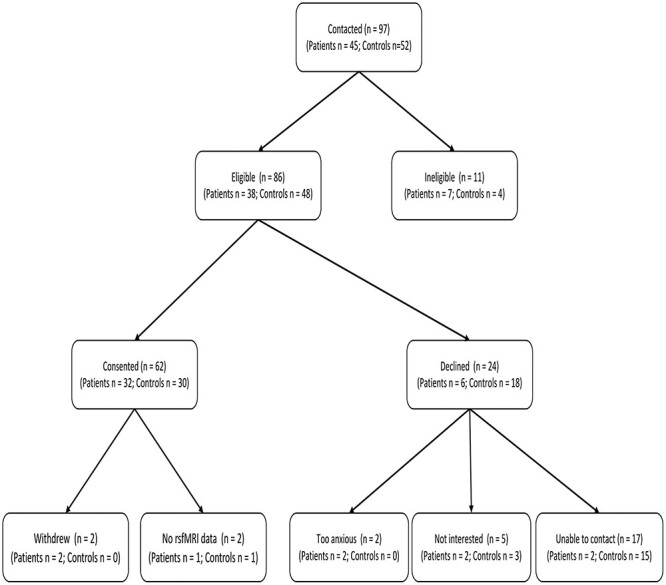
Participant flow chart.

A total of 58 participants were included in this study, of which 29 were youth with chronic headache, and 29 were age- and sex-matched healthy youths (Fig. 1). The demographic and clinical characteristics are summarized in Table 1. The types of trauma reported by patients and controls are described in Table 2. Among patients, fifteen youth (50%) had received a diagnosis of migraines, 1 (3%) had received a diagnosis of chronic headache, 7 (23%) youth had not received a diagnosis, 2 (7%) youth had post-concussive headaches, and 5 (17%) had received other diagnoses (i.e. anxiety disorder, complex regional pain syndrome, postural orthostatic tachycardia syndrome, tendonitis, and vertigo), which may or may not have been related to their headache (to be assessed, and followed by the clinic). A total of 34% (*n* = 10) of patients as compared to 76% of controls (*n* = 22) did not take medication for their pain 1-month prior to their MRI. Only 2/29 patients (6.9%) took medication daily for their headaches. The types of medications utilized by patients to manage their pain were highly variable including: analgesics, anticonvulsants, antiemetics, nonsteroidal anti-inflammatory drugs, prokinetics, selective serotonin reuptake agonists, triptans. Patients self-reported greater depressive symptoms compared to healthy controls [*t*(56) = 2.29, *P* < 0.05].

**Table 1:** Demographic and clinical characteristics of the cohort.

Demographics	Patients *n* = 29	Controls *n* = 29	*P*-value
Age, M (SD)	14.3 (2.6)	14.4 (2.6)	1.00
Sex (female), *n* (%)	20 (67.0)	20 (67.0)	1.00
Pubertal status, M (SD)	14.7 (5.2)	14.8 (4.6)	0.47
Ethnicity (White), *n* (%)	27.0 (93.1)	26.0 (89.7)	0.54
Number of headache attacks, M (SD)	16.2 (13.2)	1.4 (1.7)	<0.001
PTSS^a^, M (SD)	6.6 (10.7)	12.1 (13.6)	0.12
Anxiety symptoms, M (SD)	44.3 (16.5)	42.2 (11.5)	0.58
Depressive symptoms, M (SD)	51.0 (16.8)	42.6 (10.9)	0.03

^a^Missing data: PTSS (Patients = 5, Controls = 1); Pubertal status (Patients = 2, Controls = 2); SD = standard deviation.

**Table 2:** Types of trauma reported by patients and controls.

Types of Trauma	Patients (*n* = 25)	Controls (*n* = 29)
Direct exposure to threatened or actual (i) death, (ii) serious injury, and/or (iii) sexual violence	2 (8)	5 (17)
Witnessing threatened or actual (i) death or (ii) serious injury	2 (8)	0 (0)
Learning of threatened or actual (i) death or (ii) serious injury of a close friend or family member	3 (12)	10 (34)
Intrusive thoughts about threatened or actual (i) death or (ii) serious injury	1 (4)	2 (6)
Harassment/bullying	1 (4)	2 (6)
Parent’s divorce	1 (4)	0 (0)
Moving	0 (0)	2 (6)
Trauma not disclosed	3 (12)	0 (0)
None	13 (52)	8 (28)

Number (Percent)

### Comparison of hippocampal networks between patients and controls

**Fig. 2:**
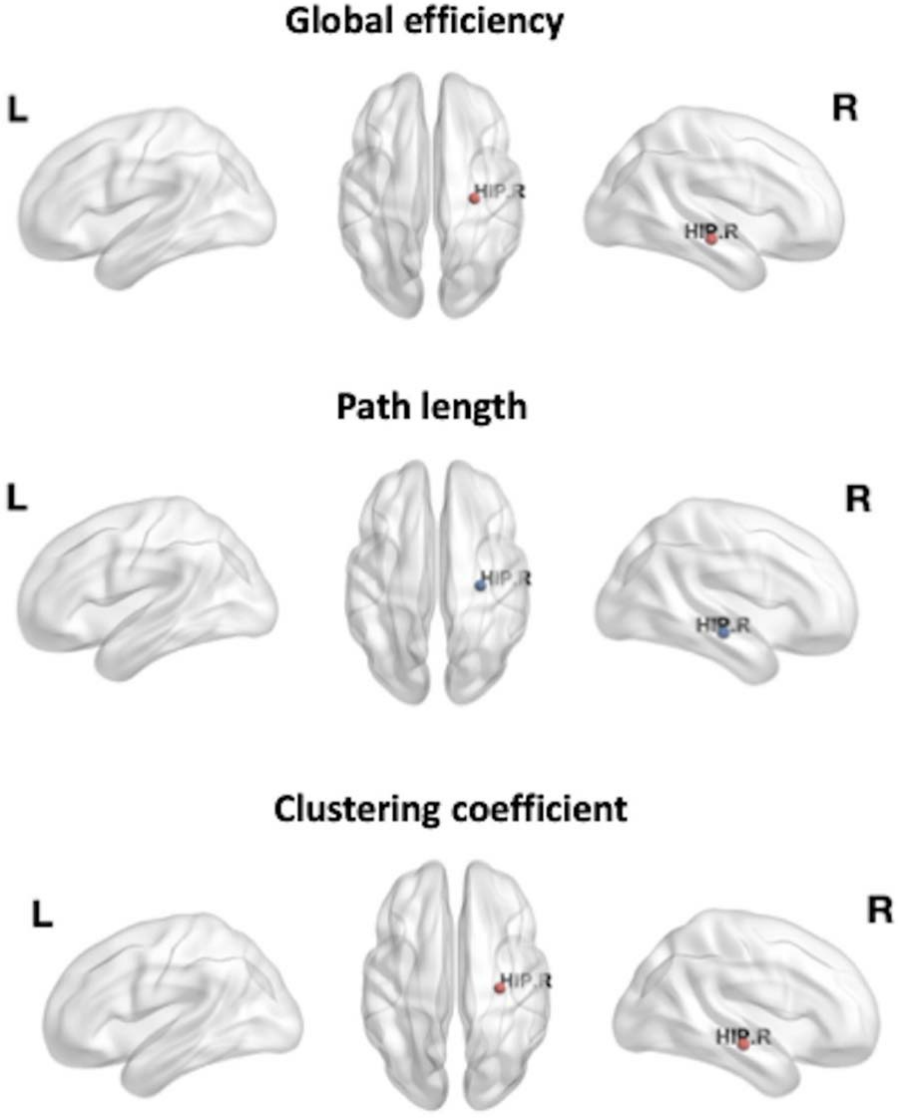
Significant group differences in right hippocampal graph theoretic metrics. Red nodes indicate larger values in patients with chronic headache compared to healthy controls. The size of the node indicates the *t* value. The statistical criterion for between-group differences was set at *P* ≤ 0.05.

Head motion (M = 1.06, standard deviation (SD) = 0.71) during the fMRI scan did not significantly differ between patients and controls [*t*(56) = 0.04, *P* = 0.97]. The overall functional connectivity did not significantly differ in between patients and controls (*P* = 0.74). Youth with chronic headache had higher right hippocampal global efficiency [*t*(56) = 2.02, *P* = 0.05, Cohen’s d = 0.53] shorter right hippocampal path length (*t*(56) = −2.19, *P* = 0.03, Cohen’s d = −0.57), and higher right hippocampal clustering coefficients (*t*(56) = 3.22, *P* = 0.002, Cohen’s d = 0.85) compared to healthy controls (Fig. 2). No differences were observed between youth with chronic headache and right hippocampal local efficiency, degree centrality, or the left hippocampal networks of controls.

### Association of hippocampal networks with headache frequency in patients

**Fig. 3:**
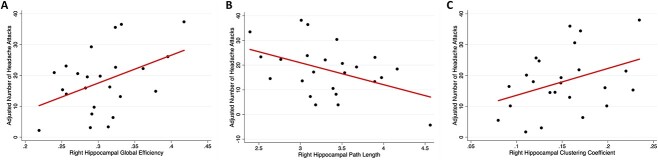
Relationship between number of headache attacks and right hippocampal networks in patients. Number of headache attacks were adjusted for child age, sex, PTSS, depressive, anxiety symptoms and brain efficiency. A) Number of headache attacks increases with greater right hippocampal global efficiency. B) Number of headache attacks decreases with greater right hippocampal path length. C) Number of headache attacks increases with greater right hippocampal clustering coefficient. Line of best fit shown.

Greater right hippocampal global efficiency (β = 0.43, *P* = 0.03; Fig. 3A), shorter right hippocampal path length (β = −0.45, *P* = 0.03; Fig. 3B), and higher right hippocampal clustering coefficient (β = 0.43, *P* = 0.05; Fig. 3C) were associated with higher headache frequency in youth with chronic headache, after controlling for child age, sex, depression, anxiety, and PTSS (see Table 3).

**Table 3:** Relationships between number of headache attacks and hippocampal networks in patients after controlling for age and sex.

		**Number of headache attacks** ***n* = 23**			
**Metrics**	**Factors**	**β**	**95% Confidence Interval**	***P***	**R** ^**2**^
**Right hippocampal global efficiency**	Depressive symptoms	1.084	0.35–1.82	0.007	0.623
	Anxiety symptoms	−0.559	−1.21 to 0.094	0.088	
	PTSS	0.225	−0.17 to 0.62	0.246	
	Pubertal status	0.941	0.084–1.798	0.033	
	Hippocampal global efficiency	0.631	0.20–1.059	0.007	
**Right hippocampal path length**	Depressive symptoms	1.105	0.357–1.853	0.007	0.619
	Anxiety symptoms	−0.538	−1.189 to 0.113	0.099	
	PTSS	0.200	0.202–0.603	0.307	
	Pubertal status	0.865	0.021–1.709	0.045	
	Hippocampus path length	−0.628	−1.059 to −0.197	0.007	
**Right hippocampal clustering coefficient**	Depressive symptoms	0.838	0.102–1.574	0.028	0.563
	Anxiety symptoms	−0.260	−0.909 to 0.389	0.406	
	PTSS	0.210	−0.224 to 0.644	0.318	
	Pubertal status	0.743	0.143–1.629	0.094	
	Hippocampus clustering coefficient	0.540	0.087–0.993	0.023	

## Discussion

The current study found that youth with chronic headache had greater depressive symptoms, higher hippocampal global efficiency, higher hippocampal clustering coefficient, and shorter hippocampal path length relative to healthy controls. Moreover, greater depressive symptoms, greater pubertal development, higher hippocampal global efficiency, higher hippocampal clustering coefficient, and shorter hippocampal path length were associated with greater headache frequency in youth with chronic headache.

Depression and chronic pain are highly comorbid, likely due in part to similarities in neurobiological underpinnings (Soltani et al. 2019). The hippocampus has previously been linked to both pain and depression (Ye et al. 2015) (Jacob et al. 2022), and in accordance with previous research, we found associations between depressive symptoms, hippocampal efficiency, and chronic headache. Our findings provide support for depression being an important factor to consider in the development of chronic headache. Moreover, it is possible the brain changes observed in our headache group may predispose youth to both depression and chronic headache.

Most studies investigating the underlying cause of headache have been conducted in the adult population. This is likely due to the fact that headache is more readily defined in the adult population and unencumbered by developmental brain changes. A study investigating whole-brain functional connectivity in adult patients with episodic cluster headache showed similarities in global efficiency between patients with headache and healthy controls (Ha and Park 2019). In contrast, Michels et al. reported a disruption in the global networks of adult patients with episodic and chronic migraine, such that migraine severity led to strong structural impairments and dysfunctional neural network configurations (Michels et al. 2021). Another study investigated topological alterations of whole-brain white matter structural networks in adults, and found that patients with headache showed higher global and local efficiencies, and lower path length in the occipital, parietal, and temporal regions of the brain compared to controls (Dai et al. 2021). This suggested that the global networks in patients were more strongly integrated, more efficient, and faster with transferring of information (Dai et al. 2021). Research focusing on the local connectivity of patients with headache has also been emerging. One study found that the local functional connectivity density of adult patients with headache decreased in the bilateral superior and middle frontal gyri, medial prefrontal cortex, precuneus, and left inferior parietal lobule, and increased in the bilateral orbital frontal gyrus, right hippocampus/parahippocampal gyrus, and cerebellum as compared to controls (Chen et al. 2017). A few other studies reported increased insular activation, as well as greater intrinsic connectivity between the insula and the default mode network in adult patients with headache as compared to controls (Bahra et al. 2001; Afridi et al. 2005; Eck et al. 2011; Xue et al. 2012). Other studies have reported the opposite findings (Kim et al. 2009; Chen et al. 2017). The differences between these studies may be explained by the progression of the disease, and consideration or lack thereof of mental health comorbidities. Similar contradictions have been found within the pediatric literature with both increases and decreases in gray matter having been reported relative to healthy controls (Webb et al. 2019). More research is needed to determine whether the observed changes in hippocampal efficiency are conserved in adults with chronic headache; and whether or not these changes persists with pain chronicity, or whether they can be modified with appropriate pain management.

The hippocampus plays a role in memory and is active in the storage and retrieval of long term explicit memories (Mansour et al. 2014), as well as facilitating and processing direct and indirect nociceptive inputs of pain (Martuscello et al. 2012). Animal studies have suggested that a reduction in the volume of the adult hippocampus as a result of reduced neurogenesis, was associated with chronic pain and depression (Mokhtari et al. 2019). However, a study examining hippocampal functional connectivity found chronic pain adult patients initially had more extensive hippocampal connectivity compared to healthy controls. As pain persisted, patients showed large decreases in functional connectivity between the hippocampus and the medial prefrontal cortex (Mutso et al. 2014). Thus, the increases in hippocampal global connectivity, higher clustering coefficient, and the shorter path length we observed in our pediatric sample with chronic headache may be temporary, and may eventually lead to the degeneration of the hippocampus in both functional connectivity and volume should their headache pain persist.

Several studies have suggested that chronic pain could be the result of maladaptive memory mechanisms, and that the long term persistence of pain after an injury could be related to the incapacity of the brain to erase a painful memory trace (Mazza et al. 2018). A recent study investigating the influence of children’s pain memories on future pain experiences found that at 5 months postoperatively, children’s pain memories were predictive of subsequent pain (Noel et al. 2017b). Another study found that younger children’s recall is less accurate and more prone to suggestibility in the context of acute and procedural pain, while older children may be more prone to pain memory distortion if they experience chronic pain (Noel et al. 2017a). In addition, children with greater levels of emotional distress are at increased risk for developing negatively biased memories of pain (i.e. remembering the pain as more intense than it was initially reported) (Noel et al. 2017a). Greater hippocampal functional connectivity and shorter path length may improve the ease with which maladaptive pain memories are stored and retrieved, thereby contributing to the chronification of pain. Moreover, greater hippocampal efficiency in patients with chronic headache may lead to the amplification of fear and painful memories via superior communication between the hippocampus associated brain structures such as the amygdala and anterior cortex (McCarberg and Peppin 2019). These relationships need to be studied longitudinally to better understand how they change over time and if they evolve as a result of the developing brain and/or the duration of chronic headache.

There are several limitations to our study. First, our study was conducted with a heterogeneous and small sample at a single time point. Future research would benefit from repeating this analysis in a larger, longitudinal, and more diverse sample of youth with a confirmed chronic headache diagnosis. This would allow for the examination of functional connectivity changes associated with disease progression versus resolution in youth.

In conclusion, youth with chronic headache demonstrated greater regional global efficiency in the hippocampus compared to healthy controls, in line with prior adult studies. Greater right hippocampal global efficiency, shorter right hippocampal path length, as well as higher right hippocampal clustering coefficient were associated with greater headache frequency in patients, after controlling for confounding factors. The hippocampus plays a significant role in memory formation and retrieval. This data thereby, supports previous findings demonstrating the importance of the hippocampus and pain memory in the chronification of pain. Further research investigating the role that memory plays in the chronification of pain could bring us a step forward in understanding the mechanisms that lead to the development and maintenance of chronic headache.

## Data Availability

Deidentified data that support the findings of this study are available from the corresponding author, upon reasonable request.
